# Comparison of Two Initial Effect-Site Concentrations of Remifentanil with Propofol During Percutaneous Vertebroplasty Under Monitored Anesthesia Care: A Randomized Controlled Study with Titration-Based Adjustment

**DOI:** 10.3390/jcm14134669

**Published:** 2025-07-01

**Authors:** Shih-Syuan Lin, Zhi-Fu Wu, Hou-Chuan Lai, Ching-Lung Ko, Ting-Yi Sun, Kun-Ting Hong, Kai-Li Lo, Tzu-Hsuan Yeh, Wei-Cheng Tseng

**Affiliations:** 1 Department of Anesthesiology, Tri-Service General Hospital, National Defense Medical Center, Taipei 114, Taiwan; xxxxx8500@gmail.com (S.-S.L.); aneswu@gmail.com (Z.-F.W.); m99ane@gmail.com (H.-C.L.); clko1168@gmail.com (C.-L.K.); kelly8014@gmail.com (K.-L.L.); 2 Department of Anesthesiology, Kaohsiung Medical University Hospital, Kaohsiung Medical University, Kaohsiung 807, Taiwan; 3 Department of Anesthesiology, Faculty of Medicine, College of Medicine, Kaohsiung Medical University, Kaohsiung 807, Taiwan; 4 Center for Regional Anesthesia and Pain Medicine, Wan Fang Hospital, Taipei Medical University, Taipei 116, Taiwan; 5 Graduate Institute of Life Sciences, National Defense Medical Center, Taipei 114, Taiwan; shelly86519@gmail.com; 6 Department of Orthopedic Surgery, Tri-Service General Hospital, National Defense Medical Center, Taipei 114, Taiwan; ericmaryptt@gmail.com; 7 Department of Neurological Surgery, Tri-Service General Hospital, National Defense Medical Center, Taipei 114, Taiwan; syndrome1028@gmail.com

**Keywords:** monitored anesthesia care, percutaneous vertebroplasty, propofol, remifentanil, target-controlled infusion

## Abstract

**Background:** Percutaneous vertebroplasty (PVP) is often performed under monitored anesthesia care (MAC) using a combination of propofol and remifentanil. However, the effects of different remifentanil effect-site concentrations (Ce) combined with propofol on perioperative outcomes in this procedure have not been reported. **Methods:** In this prospective, randomized controlled study, 80 patients scheduled for single-level PVP under MAC were enrolled. Participants were randomly assigned to receive propofol (Ce: 2.0 mcg/mL) combined with either a low (1.0 ng/mL; Group 1) or high (2.0 ng/mL; Group 2) remifentanil Ce. The primary outcome was the incidence of intraoperative patient movement; secondary outcomes included hemodynamic stability, perioperative adverse events, anesthetic consumption, frequency of dose adjustments, postoperative recovery, and anesthesia satisfaction. **Results:** Group 2 exhibited significantly fewer episodes of patient movement during the procedure and better intraoperative hemodynamic stability. Additionally, fewer upward adjustments in remifentanil infusion were observed in Group 2. Although the total propofol consumption was similar between the groups, Group 2 required a significantly lower propofol Ce to achieve adequate sedation. Surgeon satisfaction with anesthesia was also significantly higher in Group 2. **Conclusions:** Using a higher remifentanil Ce (2.0 ng/mL) in combination with propofol during PVP under MAC reduces patient movement and improves intraoperative hemodynamic stability without increasing adverse events. This regimen may thereby enhance procedural efficiency and surgeon satisfaction during vertebral interventions.

## 1. Introduction

Vertebral compression fractures (VCFs) are a growing global health concern, frequently leading to substantial pain and functional disability [1]. They are also associated with increased morbidity and mortality [2]. Osteoporosis is the primary underlying cause, particularly among the elderly, with an estimated 1.4 million new cases reported worldwide each year [3]. Other etiologies include trauma and neoplasms [1,2]. Treatment strategies for VCFs can be broadly categorized into conservative management and interventional care. Conservative treatments typically involve bed rest, analgesics and muscle relaxants, bracing, and physical therapy; however, when conservative management fails, vertebral augmentation procedures provide an effective alternative for alleviating VCF-related pain [1,2,3].

Percutaneous vertebroplasty (PVP), one of the vertebral augmentation procedures, is a minimally invasive technique commonly applied to treat VCFs, especially in the thoracic and lumbar spines. By providing strong mechanical stabilization of fractured vertebral bodies, PVP has a significant effect on rapid pain relief and improved functional outcomes [4]. Given that PVP is relatively simpler and less painful than other vertebral augmentation procedures, such as balloon kyphoplasty and lordoplasty, it can be performed under local anesthesia or monitored anesthesia care (MAC) with conscious sedation, thereby avoiding the potential complications associated with general anesthesia (GA) [5]. However, some patients cannot tolerate PVP under minimal sedation due to insufficient analgesia, prolonged procedural time, or uncooperative behavior and may require deeper sedation. Therefore, MAC during PVP has been increasingly advocated in recent decades, ensuring effective sedation and analgesia, reducing patient anxiety and distress, and minimizing intraoperative movement while maintaining hemodynamic stability [6].

Remifentanil is an ultra-short-acting phenylpiperidine opioid with potent and selective mu-opioid receptor agonist activity. It is characterized by high lipid solubility and rapid metabolism by nonspecific plasma and tissue esterases, which facilitates a rapid onset and offset of the analgesic effect [7]. Owing to its rapid onset, ease of titration, and fast recovery profile, remifentanil is considered an ideal opioid for continuous infusion to manage pain related to procedural stimulation and has gained widespread use in MAC [6]. In addition, remifentanil is often combined with propofol for MAC during various diagnostic and therapeutic procedures, and this combination appears to provide benefits such as superior analgesia and improved hemodynamic stability [8,9,10,11,12]. Despite these advantages, remifentanil is also associated with potential adverse effects, including hypotension and respiratory depression, which require prompt dose adjustments and appropriate interventions [12,13,14]. To enhance patient safety and comfort, target-controlled infusion (TCI) systems for hypnotic and analgesic agents are widely adopted during MAC [15]. These drug delivery systems calculate the infusion rate to achieve and maintain a precise plasma or effect-site concentration (Ce), based on individual pharmacokinetic parameters.

To the best of our knowledge, no study has investigated the influence of different Ce of remifentanil, in combination with propofol, on perioperative outcomes in patients undergoing PVP. To identify the effective remifentanil Ce for achieving superior outcomes, we conducted a randomized controlled study comparing two initial Ce of remifentanil in patients with VCFs scheduled for single-level PVP. This study aimed to evaluate the efficacy and safety of these two remifentanil Ce combined with propofol during PVP under MAC, and to assess their effects on pharmacological requirements, intraoperative dose adjustments, postoperative recovery profiles, and satisfaction with anesthetic management.

## 2. Materials and Methods

### 2.1. Study Population

This prospective, randomized controlled study was conducted at Tri-Service General Hospital, Taiwan. With the approval of the relevant institutional review board (TSGHIRB No. 2-107-05-163; approved on 17 March 2021) and registration in the ClinicalTrials.gov database (Registration No. NCT05876039; registered on 25 May 2023), we enrolled 80 adult patients scheduled for elective single-level PVP performed in the prone position requiring MAC between May 2023 and August 2024 and obtained written informed patient consent from all patients after providing detailed information about this study. Patients were excluded if they had any of the following features: age < 18 or > 80 years; American Society of Anesthesiologists (ASA) classification of IV or above; anatomic abnormalities of the spine; previous spine surgery; history of chronic back pain; multi-level procedure; combined surgery such as percutaneous endoscopic discectomy; active pulmonary disease; morbid obesity (body mass index [BMI] ≥ 40 kg/m^2^); pregnancy; emergency surgery; allergy to any medications used in the protocol; increased risk of aspiration; requirement for GA with tracheal intubation; or refusal to participate in this study.

### 2.2. Randomization

Using a parallel design with an allocation ratio of 1:1 and a computer-generated randomization program with a block size of 4, patients were randomized into two groups: MAC achieved through the concurrent administration of propofol (initial target Ce of 2.0 mcg/mL) and remifentanil (initial target Ce of 1.0 ng/mL for Group 1 or 2.0 ng/mL for Group 2). The randomization sequence was concealed in sequentially numbered, opaque, and sealed envelopes and was determined by an investigator who was not involved in the perioperative care. The group assignment was then announced to the anesthesiologist before surgery. The initial target Ce of remifentanil (1.0 ng/mL and 2.0 ng/mL) were chosen based on prior clinical evidence [16,17,18] and our institutional experience with elderly patients undergoing minimally invasive procedures under MAC. Concentrations above 2.0 ng/mL have been associated with a higher risk of cardiorespiratory depression, whereas 1.0 ng/mL remains an effective and conservative baseline for comparison.

### 2.3. Anesthesia and Monitoring

Upon the patient’s arrival in the operating room, standard monitoring, including three-lead electrocardiography, non-invasive blood pressure, pulse oximetry, and capnography, was instituted. Furthermore, bispectral index (BIS) monitoring (BIS^TM^ Complete 2-Channel Monitor; Medtronic, Minneapolis, MN, USA) was applied in all patients. Hemodynamic values and BIS data were recorded every 5 min. No premedication was administered before the induction of anesthesia. Participants were preoxygenated and maintained using 100% oxygen at 6 L/min via a simple face mask during the whole procedure. After preoxygenation, MAC was induced by simultaneously administering propofol at an initial Ce of 2.0 mcg/mL and remifentanil at an initial Ce of 1.0 or 2.0 ng/mL according to the group assignment delivered by a TCI pump (Orchestra^®^ Base Primea; Fresenius Kabi AG, Bad Homburg, Germany), following the Schnider and Minto models, respectively [15]. The initial propofol Ce was set at 2.0 mcg/mL based on our institutional experience with elderly patients undergoing minimally invasive procedures such as PVP under MAC. In this patient population, the effective Ce for propofol typically ranges from 2.0 to 3.0 mcg/mL. Because remifentanil has an anesthetic-sparing effect when combined with propofol, a conservative propofol Ce of 2.0 mcg/mL was selected to provide adequate sedation while minimizing the risk of adverse hemodynamic or respiratory events. When patients lost consciousness, anesthesia using TCI of propofol was titrated to maintain BIS values within the range of 40–60 [19], with upward or downward adjustments by 0.2 mcg/mL at a 30 s interval. During the maintenance of anesthesia, the Ce of remifentanil was adjusted upward or downward by 0.5 ng/mL at a 60 s interval to ensure adequate analgesia and maintain patient immobility.

In terms of hemodynamic alterations, the mean blood pressure (MBP) was kept within 30% of the baseline and the heart rate (HR) was maintained between 50 and 100 beats per minute (bpm). Intraoperatively, when patient movement occurred, or when the MBP increased by >30% from the baseline and the HR exceeded 100 bpm, the Ce of remifentanil was increased. On the contrary, TCI of remifentanil was adjusted downward when the MBP decreased by >30% from the baseline accompanied by an HR below 50 bpm. If significant hemodynamic suppression events (MBP > 30% decrease from the baseline or HR < 40 bpm for >5 min) occurred, bolus ephedrine (5–10 mg) was given for hypotension or atropine (0.5 mg) for bradycardia; conversely, any significant episodes of hemodynamic activation (MBP > 30% increase from the baseline or HR > 150 bpm for >5 min) were treated with nicardipine for hypertension or diltiazem for tachycardia. Additionally, oxygen saturation (SpO_2_) and end-tidal carbon dioxide (EtCO_2_) were monitored to assess the patients’ respiratory statuses. When apnea without desaturation (SpO_2_ ≥ 90%) occurred, jaw elevation was performed to maintain airway patency; however, positive-pressure mask ventilation (with 100% oxygen at 6 L/min) and a reduction in the Ce of remifentanil were conducted in cases of apnea with desaturation (SpO_2_ < 90%).

At the end of surgery, TCI of propofol and remifentanil was simultaneously terminated. Patients were then repositioned to the supine position. Once patients were fully awake and able to follow commands, they were transferred to the post-anesthesia care unit (PACU) for 30 min of postoperative observation and care. The criteria for discharge from the PACU were the presence of stable vital signs and an acceptable pain response (visual analogue scale [VAS] ≤ 4). Intravenous tramadol (50 mg) and droperidol (1.25 mg) were given as required for analgesic rescue and postoperative nausea and vomiting (PONV), respectively.

### 2.4. Surgical Procedure

All patients were placed in the prone position and received MAC. After proper positioning, the surgical site was meticulously sterilized, and sterile drapes were applied to establish a clean operative field. Local infiltration analgesia with 5–10 mL of 2% lidocaine was administered from the subcutaneous tissue to the pedicle of the fractured vertebra. Then, the skin was minimally incised to facilitate the passage of a trocar to access the fractured vertebra, which was approached posteriorly under imaging guidance using either a transpedicular or parapedicular technique according to the surgeon’s preference. Once the trocar was inserted into the vertebral body, polymethyl methacrylate bone cement was injected under fluoroscopic control, enabling continuous monitoring of its expansion. The injected volume of bone cement was determined based on the clinical conditions. Following the injection, the trocar was removed, and the skin incision was closed with appropriate sutures. Finally, a sterile dressing was applied to protect the surgical site postoperatively.

### 2.5. Data Collection

Patient demographics (sex, age, BMI, smoking and drinking habits, and comorbidities) and clinical data (ASA physical status, functional capacity, and surgical site) were collected from medical records. Intraoperative hemodynamic parameters (MBP, HR, respiratory rate [RR], SpO_2_, and EtCO_2_), propofol and remifentanil Ce, and BIS data at selected time points were used for analysis. Moreover, we recorded surgery and anesthesia-related variables (operation and anesthesia time, time to loss of consciousness [LOS] and recovery of consciousness [ROC], total propofol and remifentanil consumption, frequency of propofol and remifentanil pump adjustments, and volume of fluid administration) and adverse events during surgery. Pulmonary adverse events during surgery included hypoxemia (SpO_2_ < 90%), hypercapnia (EtCO_2_ > 60 mmHg), and bronchospasm, whereas cardiovascular adverse events consisted of hypotension (MBP < 60 mmHg), arrhythmias, and myocardial ischemia/infarction. The occurrence of postoperative adverse events including pulmonary and cardiovascular episodes, PONV and remifentanil-induced hyperalgesia during the first 24 h after surgery, VAS at the PACU, requirement for rescue tramadol, length of postoperative and total hospital stays, and patient and surgeon satisfaction with anesthetic management (1 = very unsatisfactory, 2 = unsatisfactory, 3 = neutral, 4 = satisfactory, 5 = very satisfactory) after 24 h postoperative observation were also documented. Postoperative pulmonary complications included atelectasis, pneumonia, and pulmonary embolism, whereas cardiovascular complications comprised hypotension (MBP < 60 mmHg), arrhythmias, and myocardial ischemia/infarction.

The primary outcome of this study was the incidence of intraoperative patient movement interfering with the procedure, used to compare two Ce of remifentanil during elective single-level PVP. Secondary outcomes included hemodynamic stability, occurrence of perioperative adverse events (e.g., pulmonary and cardiovascular episodes), total consumption of anesthetics, frequency of adjustments required for propofol and remifentanil TCI pumps during PVP, postoperative pain scores, length of hospital stay, and anesthesia satisfaction.

### 2.6. Statistical Analysis

Statistical analysis was performed with SPSS for Windows, version 23.0 (IBM SPSS Inc., Chicago, IL, USA). Continuous variables were presented as means ± standard deviations and categorical variables as numbers with percentages. The number of TCI pump adjustments and satisfaction with anesthesia as ordinal variables were presented as medians with interquartile ranges. The normality of data was checked using the Shapiro–Wilk test before statistical analysis. Normally distributed continuous variables were compared using Student’s *t*-test, whereas the Mann–Whitney U test was used for non-normally distributed variables. Categorical variables were analyzed using either the χ^2^ test or Fisher’s exact test. In addition, the numbers of adjustments for TCI pumps and levels of anesthesia satisfaction between the two groups were compared using the Mann–Whitney U test. A *p* value < 0.05 was taken to indicate a statistically significant difference.

### 2.7. Power and Sample Size

The sample size was calculated from previous data in our institute showing that the incidence of intraoperative patient movement under MAC with an initial remifentanil Ce of 1.0 ng/mL in patients undergoing single-level PVP was 45.2%. To assume a 30% reduction in the incidence of patient movement during single-level PVP with an initial remifentanil Ce of 2.0 ng/mL for MAC, at least 36 patients in each group were required with an α of 0.05 and a β of 0.2. Considering a possible dropout rate of 10%, a total of 80 patients were enrolled in this study.

## 3. Results

**Figure 1:**
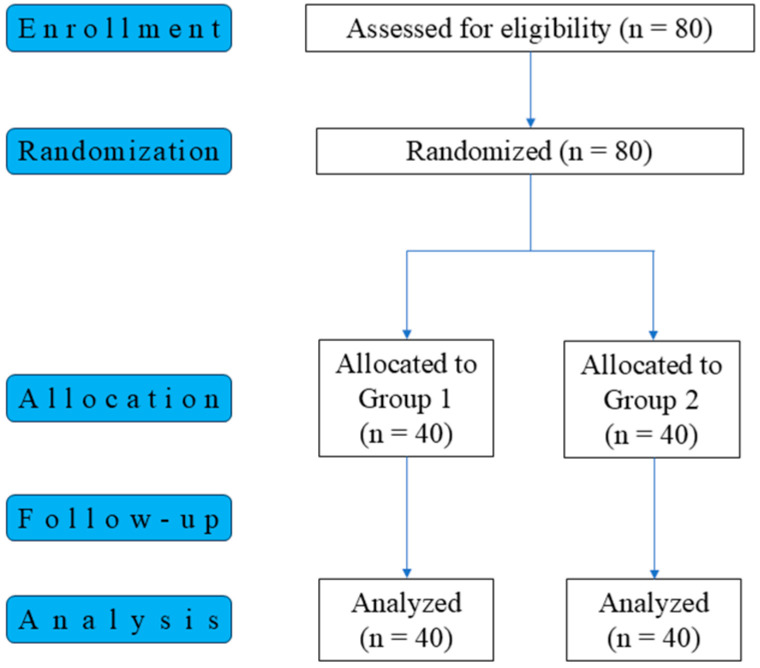
Flow diagram of patient recruitment according to the study protocol. Group 1, 1.0 ng/mL of initial effect-site concentration of remifentanil group; Group 2, 2.0 ng/mL of initial effect-site concentration of remifentanil group.

A total of 80 patients were initially recruited for this study (Figure 1). All patients met the inclusion criteria and were randomly assigned to the two groups. No participants were excluded after randomization. Ultimately, all enrolled patients completed this study, and their data were analyzed.

### 3.1. Demographic Data and Surgical Information

Patient demographics and surgical characteristics, including sex, age, habitus, tobacco and alcohol use, underlying disease, ASA class, functional status, and procedure site, were not significantly different between the groups (Table 1).

### 3.2. Surgery and Anesthesia-Related Details

Patients in the two groups had similar operation and anesthesia times (Table 2). There was a trend toward a shorter time to achieve LOC for patients in Group 2 (2.69 ± 1.26 min) than those in Group 1 (3.19 ± 1.20 min), although the difference did not reach statistical significance (*p* = 0.074); conversely, the time to attain ROC was comparable between the groups (Table 2). In addition, the administered fluid volume between the groups was comparable (Table 2).

Regarding the consumption of anesthetics during surgery, the propofol consumption did not differ significantly between the groups, whereas the remifentanil consumption was significantly higher in Group 2 (208.50 ± 78.61 mcg) compared to Group 1 (141.40 ± 75.25 mcg; *p* < 0.001) (Table 2). Even though the difference was not statistically significant (*p* = 0.069), patients in Group 1 seemed to require more frequent upward adjustments of the propofol TCI pump (2.0 [1.0–3.0]) than those in Group 2 (2.0 [1.0–2.0]). Similarly, there were significantly more frequent upward adjustments of the remifentanil TCI pump in Group 1 (1.0 [0.0–1.0]) compared to Group 2 (0.0 [0.0–1.0]; *p* < 0.001). The frequency of downward adjustments of propofol was statistically similar between the groups. While the number of downward adjustments of remifentanil appeared lower in Group 1 (1.0 [1.0–1.0]) compared to Group 2 (1.0 [1.0–2.0]), the difference did not reach statistical significance (*p* = 0.063). No significant differences were observed between the groups for total anesthetic adjustments using TCI pumps (Table 2).

### 3.3. Hemodynamic and Sedation Profiles During Surgery

**Figure 2:**
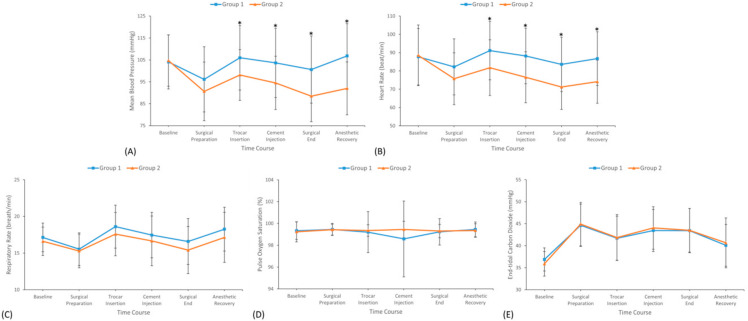
Cardiovascular parameters: (**A**) mean blood pressure, (**B**) heart rate; and respiratory variables: (**C**) respiratory rate, (**D**) oxygen saturation, (**E**) end-tidal carbon dioxide during surgery. Data were expressed as mean ± standard deviation. Group 1, 1.0 ng/mL of initial effect-site concentration of remifentanil group; Group 2, 2.0 ng/mL of initial effect-site concentration of remifentanil group; *, *p* < 0.05.

Patients in Group 2 had a significantly more stable MBP compared to those in Group 1 from the start of surgery until the recovery from anesthesia (Figure 2A). Likewise, there were significantly more controlled HRs in Group 2 than Group 1 (Figure 2B). As for RR, SpO_2_, and EtCO_2_, these variables did not differ significantly between the groups (Figure 2C–E).

**Figure 3:**
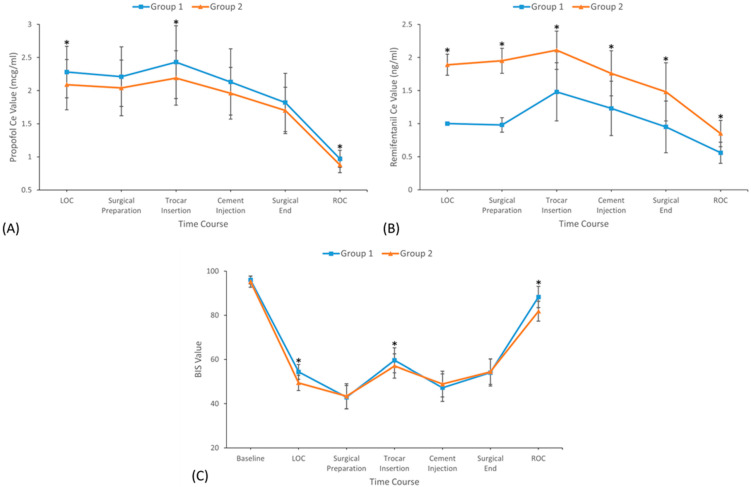
(**A**) Propofol Ce, (**B**) remifentanil Ce, and (**C**) BIS values during surgery. Data were expressed as mean ± standard deviation. BIS, bispectral index; Ce, effect-site concentration; Group 1, 1.0 ng/mL of initial effect-site concentration of remifentanil group; Group 2, 2.0 ng/mL of initial effect-site concentration of remifentanil group; *, *p* < 0.05.

The propofol Ce were significantly lower in Group 2 compared to Group 1 at specific time points, including LOC, trocar insertion, and ROC (Figure 3A). The difference may result from the anesthetic-sparing effect of remifentanil, which was associated with significantly higher Ce of remifentanil in Group 2 than Group 1 (Figure 3B). Furthermore, patients in Group 2 exhibited a significantly deeper sedation state, reflected by BIS values at specific time points (LOC, trocar insertion, and ROC) compared to those in Group 1 (Figure 3C), despite the anesthetic-sparing effect of higher remifentanil Ce.

### 3.4. Intraoperative Adverse Events

With respect to intraoperative adverse events, there were no significant differences in pulmonary or cardiovascular complications between the groups (Table 2). No patient experienced hemodynamic crises requiring the discontinuation of anesthetic agents. Vasoactive agents were administered infrequently and only in low doses when required. Except for ephedrine, no other vasoactive drugs were used. Among patients who received ephedrine, none required more than 20 mg. There were no significant differences in the use of vasoactive agents between the groups (Table 2). Notably, the incidence of patient movement interrupting the procedure was significantly higher in Group 1 (42.5%) compared to Group 2 (10.0%; *p* = 0.001) (Table 2).

### 3.5. Postoperative Assessment and Satisfaction

No difference between the groups was observed in postoperative adverse events including pulmonary or cardiovascular complications, PONV, and remifentanil-induced hyperalgesia during the first 24 h after surgery (Table 3). One postoperative pulmonary adverse event was reported in each group. Both incidents occurred in the PACU and involved transient desaturation (SpO_2_: 85–90%) without associated symptoms. In both cases, SpO_2_ levels improved to > 90% with oxygen supplementation via a nasal cannula at 3 L/min, requiring no further intervention. Neither event led to advanced respiratory complications or prolonged PACU stay. Although patients in Group 1 had a significantly higher VAS at the PACU (1.30 ± 0.97) than those in Group 2 (0.90 ± 0.38; *p* = 0.018), both scores fell within the minimal to mild pain range. Thus, there was no difference in the immediate need for rescue tramadol between the groups (Table 3). Neither the postoperative nor the total hospital stay differed significantly between the groups (Table 3).

All patients’ appraisals of MAC were at least satisfactory, and there was similar satisfaction with anesthesia between the groups (Table 3). However, surgeons reported significantly higher satisfaction with anesthesia during surgery for patients in Group 2 (4.5 [4.0–5.0]) compared to those in Group 1 (4.0 [4.0–4.0]; *p* < 0.001) (Table 3).

## 4. Discussion

This randomized controlled study compared the effects of two initial Ce of remifentanil (1.0 vs. 2.0 ng/mL), in combination with propofol, on perioperative outcomes in patients undergoing single-level PVP under MAC. The findings demonstrated that a higher remifentanil Ce (2.0 ng/mL) was associated with reduced intraoperative movement, favorable hemodynamic stability, and better surgeon satisfaction, without an increased incidence of perioperative complications. Although few studies have investigated the safety and efficacy of remifentanil during PVP under MAC [20,21], none have evaluated its use in conjunction with continuous propofol infusion. Given the short procedural duration and brief hospitalization following PVP, anesthetic agents should be selected to provide adequate sedation, analgesia, anxiolysis, and amnesia, along with control of patient movements, while allowing for rapid emergence and minimizing the risk of adverse events. The remifentanil–propofol combination appeared to fulfill these requirements in the present study, consistent with its reported efficacy in other procedural settings [22,23,24,25,26].

Patient movement during MAC presents a major challenge for anesthesiologists, as it can disrupt surgical procedures and increase the risk of patient injury. In our study, a higher remifentanil Ce significantly reduced the occurrence of patient movement and improved surgeon satisfaction with anesthesia, consistent with results from previous research involving other minor procedures [17,18]. Minimizing patient movement during PVP enables precise maneuvers, enhances procedural efficiency, and lowers the risk of trocar displacement or bone cement misplacement. Furthermore, improved intraoperative positioning stability may decrease imaging interruptions and subsequently limit radiation exposure for both surgical and anesthesia teams. Notably, prior studies have shown that higher Ce or infusion rates of remifentanil are associated with an increased incidence of hemodynamic and respiratory compromise [17,18,27], which were not observed in our findings. The discrepancies in cardiopulmonary adverse events may be attributed to heterogeneity in patient populations and surgical procedures, inconsistencies in the choice of sedative agents, and, most importantly, differences in monitoring practices, which were more comprehensive in our study. Therefore, further studies are warranted to clarify the influence of monitoring intensity and anesthetic protocols on the patient safety profile of remifentanil.

Hemodynamic stability, a key determinant of successful MAC, is essential for minimizing intraoperative risks such as myocardial ischemia or cerebrovascular events, particularly in elderly patients or those with comorbidities [28]. A stable intraoperative course also reduces the need for vasoactive medications, thereby simplifying anesthetic management and enhancing overall patient safety. The use of remifentanil during MAC has been associated with an attenuation of stress-induced hemodynamic fluctuations [11,23], likely due to its potent analgesic and sympatholytic properties. Our findings demonstrated that a remifentanil Ce of 2.0 ng/mL, compared to 1.0 ng/mL, more effectively blunted noxious responses to procedural stimuli, resulting in significantly greater hemodynamic stability, with fewer fluctuations in MBP and HR throughout the PVP procedure. Hemodynamic stability in our study was primarily evaluated through intraoperative MBP and HR profiles, while the use of rescue vasoactive agents was documented as supplementary evidence. Rescue ephedrine was administered infrequently and at low doses (≤20 mg), with no significant differences observed between the groups. No patient required atropine or other vasoactive agents, and no hemodynamic crises necessitating discontinuation of anesthesia occurred. These results further support the observed differences in intraoperative hemodynamic stability and underscore the safety of the anesthetic regimens used. Although certain adjuvants used in MAC have demonstrated superior hemodynamic control and lower complication rates compared to remifentanil in specific patient populations and procedural contexts [12,21,29,30], their use may contribute to prolonged recovery times [12,29]. Thus, given the variability in patient physiology and procedural complexity, anesthetic management should be tailored to individual needs rather than relying on a single standardized agent. It is worth noting that no adjuvant agents were used in this study; only remifentanil and propofol were administered, which may have helped avoid prolonged sedation and facilitate faster recovery.

In this study, MAC was conducted using a combination of propofol and remifentanil administered via TCI, with effect-site titration based on the Schnider and Minto models, respectively. Although the total numbers of TCI pump adjustments for both propofol and remifentanil were statistically similar between the groups, patients receiving a higher remifentanil Ce (2.0 ng/mL) required significantly fewer upward dose adjustments of remifentanil compared to those in the lower Ce group. While the absolute difference in adjustment frequency was relatively small, it may still represent clinically meaningful benefits in the context of a short-duration procedure such as PVP. Each titration typically reflects a physiological perturbation; therefore, a reduced need for adjustment may indicate a more stable intraoperative course with less demand for intervention. This finding suggests that an initial remifentanil Ce of 2.0 ng/mL was associated with more stable analgesia during PVP. Moreover, remifentanil demonstrated an anesthetic-sparing effect, as the higher-Ce group required significantly lower propofol Ce to maintain adequate sedation. This observation aligns with a previous randomized controlled trial, which reported that increasing remifentanil concentrations significantly reduced the Ce of propofol required for sedation [31]. However, the total propofol consumption remained comparable between the groups in our study, probably due to the use of BIS-guided titration. Our results also demonstrated similar times to LOC and ROC regardless of the remifentanil Ce. Notably, the mean time to achieve LOC in both groups was markedly shorter than that reported in patients receiving propofol alone for drug-induced sleep endoscopy [26]. Even though there were differences across studies in variables such as age and BMI, our findings still support a potential synergistic effect of remifentanil in facilitating sedation. As for the time to achieve ROC, the remifentanil concentration did not significantly delay emergence in our study, likely due to its pharmacokinetic properties and the short duration of infusion; however, patients in the higher-Ce group exhibited a relatively deeper sedation status during the recovery phase, which may help attenuate hemodynamic fluctuations when repositioning to supine and transferring to the PACU.

TCI is an advanced drug delivery system that allows the precise administration of intravenous agents such as propofol and remifentanil by targeting either plasma concentrations or Ce, based on validated pharmacokinetic models [15]. Through real-time adjustment of infusion rates, TCI offers enhanced control, stability, and reproducibility in sedation and anesthesia, supporting both clinical application and pharmacologic research [32]. Moerman et al. demonstrated that remifentanil administered via TCI for patients undergoing elective colonoscopy not only reduced total propofol requirements but also resulted in a significantly lower incidence of respiratory adverse events compared to manual infusion techniques [33]. Similarly, Tănase et al. reported that the addition of remifentanil at a Ce of 1.0 ng/mL to a propofol-based TCI regimen during drug-induced sleep endoscopy significantly reduced the required propofol Ce for adequate sedation and shortened the time to procedural readiness, without increasing the risk of hypoxemia, cardiovascular instability, or cough reflex [26]. Collectively, these findings highlight the pharmacodynamic advantages of dual-agent TCI using remifentanil and propofol, demonstrating an improved procedural efficiency and sedation safety, while reducing the risks of overshoot and drug accumulation inherent to manual infusion techniques.

This study had several limitations. First, it was conducted at a single medical center, and further large-scale, multicenter studies are needed to validate our findings. Second, the anesthesiologists performing MAC were not blinded to the group allocation, which may have introduced bias in subjective outcomes (e.g., decisions to titrate Ce or movement reporting). Third, this study only investigated two specific Ce of remifentanil (1.0 and 2.0 ng/mL). A more comprehensive dose–response analysis involving additional concentration levels would be necessary to determine the optimal Ce selection. Fourth, the study design incorporated age-specific considerations, indicating that our results may not be directly applicable to adolescents and extremely elderly patients (aged > 80 years). Lastly, since all enrolled patients were of Taiwanese ethnicity, the lack of ethnic diversity may limit the generalizability of our findings to more heterogeneous or international populations.

## 5. Conclusions

This study supports the use of a higher remifentanil Ce (2.0 ng/mL) in combination with propofol for single-level PVP under MAC, demonstrating fewer intraoperative disruptions, more stable hemodynamic responses, and greater surgeon satisfaction, without increasing the incidence of perioperative complications. These conclusions should be interpreted within the limitations of the study design and outcome measures. Further investigations involving diverse patient populations and procedural settings are warranted to validate these findings and extend their clinical applicability.

## Figures and Tables

**Table 1:** Patient demographics and surgical characteristics.

Variables	Group 1 (n = 40)	Group 2 (n = 40)	*p* Value
Sex, male/female (n [%])	9/31 (22.5%/77.5%)	12/28 (30.0%/70.0%)	0.446
Age (years old)	70.03 ± 7.46	68.50 ± 7.87	0.377
BMI (kg/m^2^)	24.42 ± 4.34	23.89 ± 4.34	0.587
Cigarette smoking, n (%)	5 (12.5%)	6 (15.0%)	0.745
Alcohol consumption, n (%)	4 (10.0%)	1 (2.5%)	0.166
Comorbidities, n (%)			
Hypertension	23 (57.5%)	24 (60.0%)	0.820
Diabetes mellitus	13 (32.5%)	8 (20.0%)	0.204
Hyperlipidemia	14 (35.0%)	16 (40.0%)	0.644
Cardiac disease	7 (17.5%)	5 (12.5%)	0.531
Respiratory disease	3 (7.5%)	1 (2.5%)	0.305
Neurological disease	3 (7.5%)	5 (12.5%)	0.456
Hepatic disease	3 (7.5%)	3 (7.5%)	1.000
Renal disease	3 (7.5%)	2 (5.0%)	0.644
Thyroid disease	3 (7.5%)	2 (5.0%)	0.644
Rheumatic disease	3 (7.5%)	3 (7.5%)	1.000
Psychiatric disease	4 (10.0%)	6 (15.0%)	0.499
Malignancy	8 (20.0%)	10 (25.0%)	0.592
ASA class, II/III (n [%])	25/15 (62.5%/37.5%)	19/21 (47.5%/52.5%)	0.178
≥4 METs, n (%)	37 (92.5%)	35 (87.5%)	0.456
Surgical site, T/L spine (n [%])	17/23 (42.5%/57.5%)	17/23 (42.5%/57.5%)	1.000

Data were expressed as mean ± standard deviation and case number (percentage). ASA, American Society of Anesthesiologists; BMI, body mass index; Group 1, 1.0 ng/mL of initial effect-site concentration of remifentanil group; Group 2, 2.0 ng/mL of initial effect-site concentration of remifentanil group; MET, metabolic equivalent; T/L spine, thoracic/lumbar spine.

**Table 2:** Intraoperative phase profile.

Variables	Group 1 (n = 40)	Group 2 (n = 40)	*p* Value
Operation time (min)	31.80 ± 14.54	30.73 ± 14.14	0.738
Anesthesia time (min)	53.33 ± 19.90	48.03 ± 16.60	0.200
Time to LOC (min)	3.19 ± 1.20	2.69 ± 1.26	0.074
Time to ROC (min)	4.64 ± 1.15	4.52 ± 1.00	0.606
Propofol consumption (mg)	328.63 ± 135.61	281.13 ± 111.84	0.091
Propofol pump adjustment (n)			
Total times	5.0 (4.5–7.5)	4.5 (4.0–6.0)	0.101
Upward times	2.0 (1.0–3.0)	2.0 (1.0–2.0)	0.069
Downward times	3.0 (3.0–4.0)	3.0 (3.0–4.0)	0.603
Remifentanil consumption (mcg)	141.40 ± 75.25	208.50 ± 78.61	<0.001
Remifentanil pump adjustment (n)			
Total times	2.0 (1.0–3.0)	2.0 (1.0–2.0)	0.193
Upward times	1.0 (0.0–1.0)	0.0 (0.0–1.0)	<0.001
Downward times	1.0 (1.0–1.0)	1.0 (1.0–2.0)	0.063
Fluid administration (mL)	202.50 ± 80.02	197.50 ± 81.61	0.783
Intraoperative adverse events, n (%)			
Pulmonary episodes	2 (5.0%)	0 (0.0%)	0.152
Cardiovascular episodes	3 (7.5%)	3 (7.5%)	1.000
Patient movement	17 (42.5%)	4 (10.0%)	0.001
Ephedrine administration, n (%)	3 (7.5%)	3 (7.5%)	1.000
Atropine administration, n (%)	0 (0.0%)	0 (0.0%)	1.000

Data were expressed as mean ± standard deviation, median (interquartile range) and case number (percentage). Group 1, 1.0 ng/mL of initial effect-site concentration of remifentanil group; Group 2, 2.0 ng/mL of initial effect-site concentration of remifentanil group; LOC, loss of consciousness; ROC, recovery of consciousness.

**Table 3:** Postoperative phase profile.

Variables	Group 1 (n = 40)	Group 2 (n = 40)	*p* Value
Postoperative adverse events, n (%)			
Pulmonary episodes	1 (2.5%)	1 (2.5%)	1.000
Cardiovascular episodes	0 (0.0%)	0 (0.0%)	1.000
Postoperative nausea and vomiting	1 (2.5%)	2 (5.0%)	0.556
Remifentanil-induced hyperalgesia	0 (0.0%)	0 (0.0%)	1.000
VAS at PACU	1.30 ± 0.97	0.90 ± 0.38	0.018
Rescue tramadol, n (%)	3 (7.5%)	0 (0.0%)	0.077
Postoperative hospital stay (day)	1.85 ± 1.89	1.88 ± 2.28	0.958
Total hospital stay (day)	3.58 ± 3.15	4.28 ± 5.49	0.486
Patient satisfaction	4.0 (4.0–5.0)	4.0 (4.0–5.0)	1.000
Surgeon satisfaction	4.0 (4.0–4.0)	4.5 (4.0–5.0)	<0.001

Data were expressed as mean ± standard deviation, median (interquartile range), and case number (percentage). Satisfaction was evaluated using a 5-point Likert scale (1 = very unsatisfactory, 2 = unsatisfactory, 3 = neutral, 4 = satisfactory, 5 = very satisfactory). Group 1, 1.0 ng/mL of initial effect-site concentration of remifentanil group; Group 2, 2.0 ng/mL of initial effect-site concentration of remifentanil group; PACU, post-anesthesia care unit; VAS, visual analogue scale.

## Data Availability

The data analyzed in this study are available from the corresponding author on reasonable request.
